# New insight into the residual inactivation of *Microcystis aeruginosa* by dielectric barrier discharge

**DOI:** 10.1038/srep13683

**Published:** 2015-09-08

**Authors:** Lamei Li, Hong Zhang, Qing Huang

**Affiliations:** 1 Key Laboratory of Ion Beam Bio-engineering, Institute of Biotechnology and Agriculture Engineering, Hefei Institutes of Physical Science, Chinese Academy of Sciences, 230031.; 2 The University of Science and Technology of China, Hefei, Anhui, 230031, P.R. China.

## Abstract

We report the new insight into the dielectric barrier discharge (DBD) induced inactivation of *Microcystis aeruginosa*, the dominant algae which caused harmful cyanobacterial blooms in many developing countries. In contrast with the previous work, we employed flow cytometry to examine the algal cells, so that we could assess the dead and living cells with more accuracy, and distinguish an intermediate state of algal cells which were verified as apoptotic. Our results showed that the numbers of both dead and apoptotic cells increased with DBD treatment delay time, and hydrogen peroxide produced by DBD was the main reason for the time-delayed inactivation effect. However, apart from the influence of hydrogen peroxide, the DBD-induced initial injures on the algal cells during the discharge period also played a considerable role in the inactivation of the DBD treated cells, as indicated by the measurement of intracellular reactive oxygen species (ROS) inside the algal cells. We therefore propose an effective approach to utilization of non-thermal plasma technique that makes good use of the residual inactivation effect to optimize the experimental conditions in terms of discharge time and delay time, so that more efficient treatment of cyanobacterial blooms can be achieved.

Water eutrophication has become a serious global problem, which can cause harmful cyanobacterial blooms^1^,^2^. Among the blooming cyanobacteria, *Microcystis aeruginosa* produces and releases toxins which pose constant threat to our environment and human health. Therefore, there is an urgent need to develop efficient techniques to control and reduce the adverse impact of blooms. To suppress or remove cyanobacteria blooms, various methods have been adopted such as chemical treatment^3^,^4^,^5^, UV radiation^6^,^7^, ultrasound irradiation^8^,^9^,^10^, electron beam irradiation^11^ and non-thermal plasma oxidation technology^2^,^12^,^13^,^14^,^15^,^16^. However, the chemical methods such as excessive use of algaecides can lead to secondary pollution, while the physical methods such as UV radiation, sonication and electron-beam irradiation have the limitation for bloom control with high efficiency or on a large scale.

As an emerging technology, plasma oxidation technology has received special attention and been applied extensively in wastewater treatment^17^. Being one of advanced oxidation processes (AOPs), it possesses both the physical and chemical processing traits, and exhibits the advantages in simpler equipment, easier operation, higher energy efficiency and environmental compatibility^2^,^18^,^19^. Among various plasma techniques, the non-thermal atmospheric dielectric barrier discharge (DBD) may be a most promising one. In fact, non-thermal DBD has been widely applied in inactivation of pathogen^20^,^21^, chemical synthesis^22^, film deposition^23^, material surface modification^24^ and etc. When plasma takes place either over the solution surface or in the solution, a variety of physical and/or chemical processes are initiated. Besides the energetic particles including negatively charged electrons and positively charged ions, the accompanying intrinsic UV emission and impact waves, chemically active substances such as hydrogen, oxygen, hydrogen peroxide and active radicals such as hydroxyl radical, hydroperoxyl radical, oxygen radical and hydrogen radical are produced during the plasma discharge^2^,^25^,^26^,^27^,^28^. These active chemical species can inactivate algal cells as reported by our previous study^2^ and other studies^1^,^12^,^15^.

However, to promote DBD application in remediation of algae contaminated wastewater, more research should be rendered to address the underlying basic questions and improve the efficiency of utilization of this technique. First, we noticed that in the previous studies, the inactivation efficiency of algal cells treated by discharge plasma oxidation were calculated by the absorbance at 680 nm which is attributed to the absorption band of *M. aeruginosa* cell suspensions, mostly from chlorophyll^15^. However, when the *M. aeruginosa* cells are damaged by discharge plasma oxidation, the cell membrane are ruptured and the pigments inside the algal cells are released into the suspension. These pigments may also have the absorbance at 680 nm. This may bring considerable error in the evaluation of the inactivation efficiency, and therefore a more accurate and reliable assessment method is required for an in-depth research. Second, the mechanism for the algae inactivation should be scrutinized more carefully with the identification of different inactivation pathways. It is known that during the discharge plasma oxidation, varied reactive species are produced in the algal suspensions which lead to algae inactivation^29^ and among these active radicals, hydroxyl radical plays the major role^1^,^2^,^14^,^15^. However, it is also known that treatment with sole hydrogen peroxide can also lead to the inactivation of algae significantly^30^. Since DBD produces hydrogen peroxide as well, this H_2_O_2_ induced inactivation of algal cells should also be taken into account for the evaluation of the overall efficiency of DBD treatment, which is, however, has been largely ignored in the previous research. Besides, discrimination of different inactivation ways such as apoptosis and necrosis can facilitate us to gain a better understanding of the algal inactivation mechanism. Third, although it has been observed that the inactivation rate still increases with delay time after the plasma treatment (so called residual inactivation effect)^1^,^14^,^15^, formerly researchers focused more on the investigation of the influence of some electrical parameters such as power and voltage on the inactivation effect to determine the optimal experimental conditions^1^. Now, with the acknowledgement of the residual inactivation effect, it is also intriguing for us if we can make use of this residual effect to improve the efficiency of DBD treatment; and if yes, how can we make the best use of it?

For these reasons, we therefore initiated this study, in which we employed flow cytometry to evaluate the inactivation efficiency of algal cells treated by DBD, and investigated the mechanism for the inactivation of algal cells by discriminating the dead and apoptotic cells and scrutinizing the residual inactivation effect caused by both hydrogen peroxide and intracellular ROS. Moreover, based on the analysis of residual inactivation effect, we attempted to establish a method for providing a useful guide for determination of the optimal experimental conditions regarding discharge and delay times, so that we could achieve the best utilization of the residual inactivation effect and improve the efficiency of plasma technology in bloom control.

## Results

**Figure 1:**
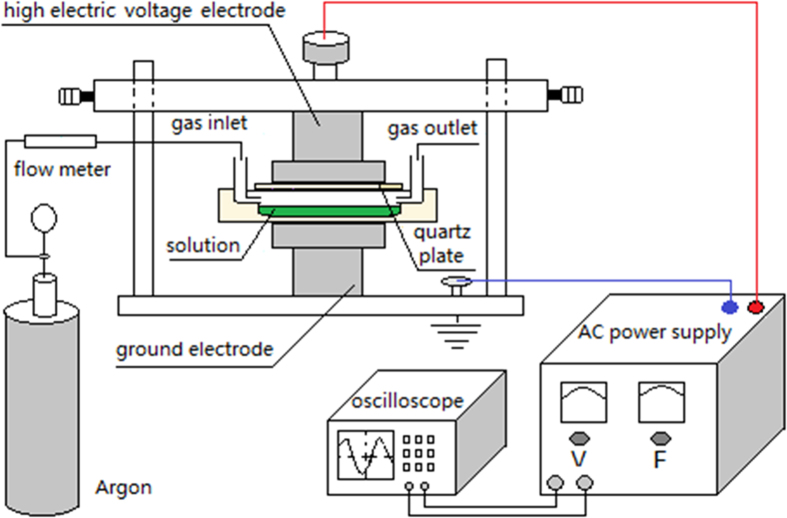
Schematic diagram of the experimental apparatus.

**Figure 2:**
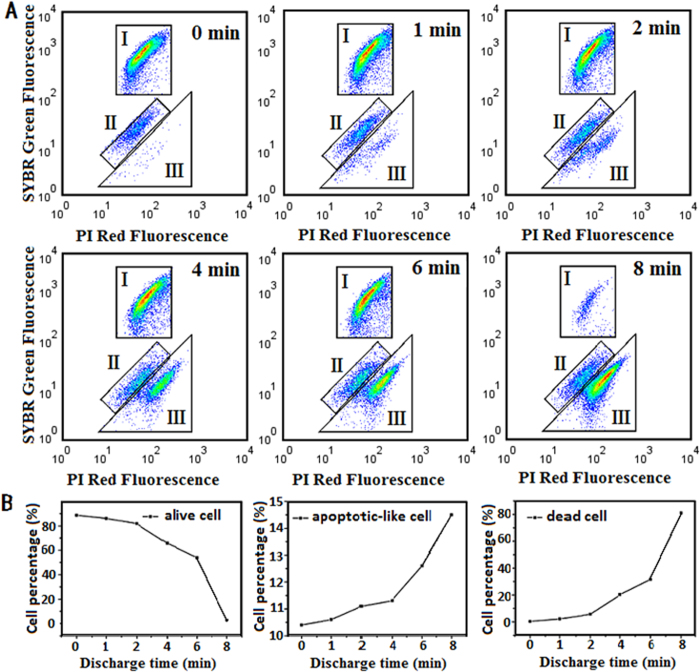
The identification of three states of the algal cells after DBD treatment without delay. (**A**) The flow cytometry results of the DBD treated cells; (**B**) The statistical graph of the flow cytometry results change with discharge time. In the flow cytometry graphs, three regions are indicated: I- live cells; II-apoptotic-like cells; III- dead cells.

We conducted the DBD experiments using the set-up as shown in Fig. 1. We changed the voltage from 10 to 20 kV, and it was observed that the inactivation rate increased with the discharge voltage, and when the applied voltage was over 16 kV, the inactivation rate was raised significantly (Supplementary Fig. S1). The inactivation effect is also dependent on the working gas of DBD, as we observed that air-DBD was more efficient in activation of *M. aeruginosa* than argon-DBD (Supplementary Fig. S2). For convenience, in this paper unless otherwise mentioned we only present the data obtained from atmospheric argon-DBD with applied voltage at 16 kV. After the DBD treatment, we employed flow cytometry to examine the DBD-treated algal cells, which were labeled with SYBR green I and/or propidium iodide (PI) for discriminating the cells with intact and damaged membrane, respectively. As seen in Fig. 2, the number of living cells decreases while the number of dead cells increases with discharge time. Strikingly, with the facility of flow cytometry, we discovered that apart from the living and dead cells, there also existed the third state of cells different from the states of living cells and dead cells, and the population of these cells also increased with plasma discharge time.

**Figure 3:**
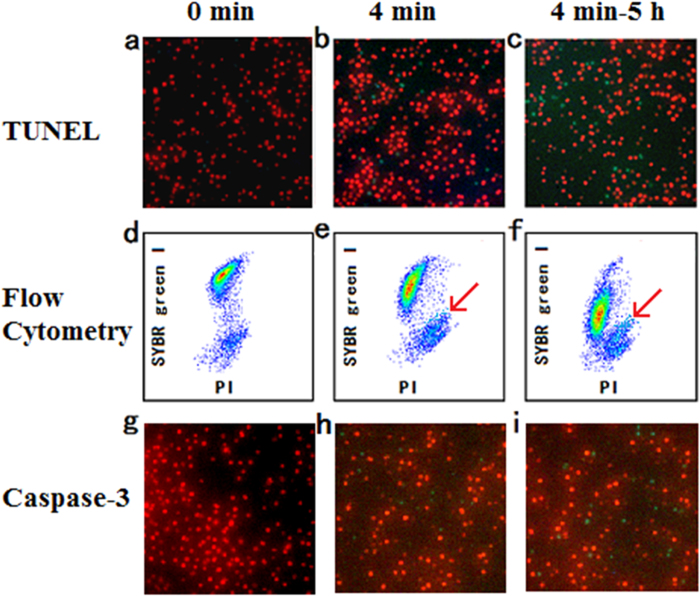
Detection of the apoptosis process after DBD plasma discharge. (**a**–**c**) TUNEL assay, (**d**–**f**) Flow cytometry assay for inactivation rate, (**g**–**i**) Caspase-3 activity assay. a,d,g: Untreated cells, b,e,h: DBD treated for 4 minutes, c,f,i: 5 hours after DBD treated for 4 minutes. The region which the red arrows point to shows the apoptotic cells.

We speculated that these cells were apoptotic cells or apoptotic-like cells^30^. To verify this, we then conducted the apoptosis assays, namely, terminal deoxynucleotidyl transferase labeling (TUNEL) assay and caspase-3 assay, and the results are illustrated in Fig. 3. The middle panel of Fig. 3 presents the result of flow cytometry, demonstrating three different states of the DBD treated cells. Correspondingly, the upper and lower panels of Fig. 3 are the fluorescent images of the algal cells: the red spots in the images are living and dead cells with auto-fluorescence, while the green spots are apoptotic cells labeled with the fluorescent dye by the apoptosis kits. It has been reported that sole H_2_O_2_ treatment can lead to apoptosis of algal cells, and indeed, this was also checked and verified by our own experiments (Supplementary Fig. S3 and Fig. S4). Therefore, compared with the cells without DBD treatment (Fig. 3a,d,g), both the TUNEL assay (Fig. 3a–c) and caspase-3 assay (Fig. 3g–i) confirm that DBD treatment can indeed lead to apoptosis of algae. In addition, we also examined the algal cells 5 hours later after the DBD treatment (Fig. 3c,f,i). Compared with the measurement from the DBD treated sample without delay (or the delay time is substantially short enough), the number of apoptotic cells is larger, indicating that elongation of both discharge time and delay time can make the remarkable contribution to apoptosis of algae.

**Figure 4:**
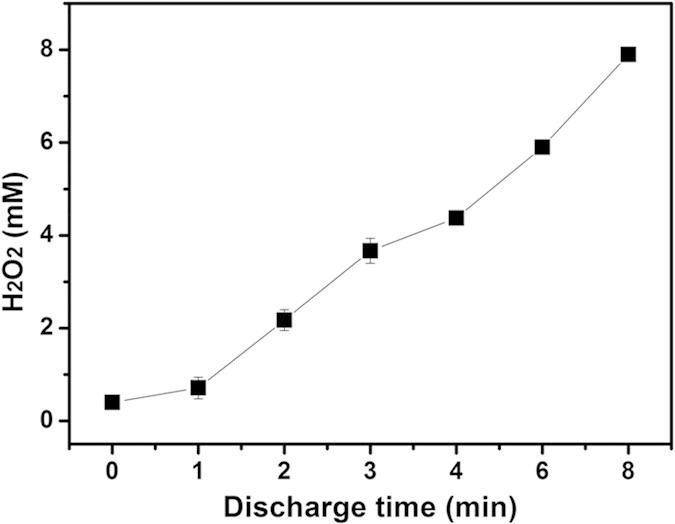
H_2_O_2_ concentration after DBD treatment for different discharge times. The measurements were repeated for three times.

**Figure 5:**
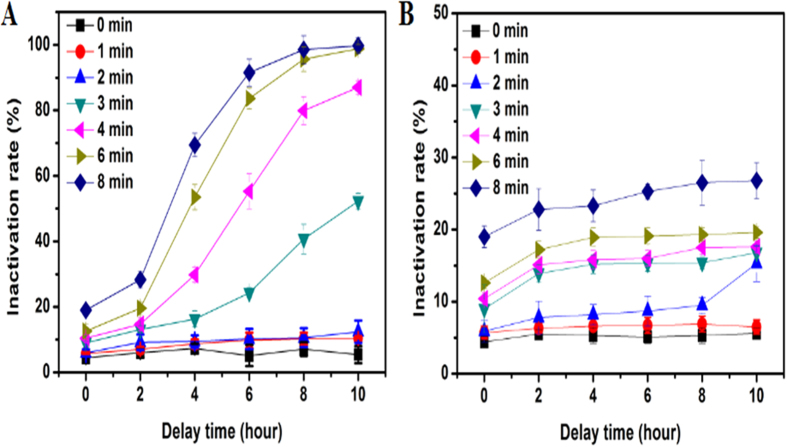
The residual inactivation rates of different *M. aeruginosa* samples with different discharged and delay times. (**A**) Measurements of the samples containing both algal cells and the DBD produced H_2_O_2_; (**B**) Measurements of the samples discarding the supernatant and re-suspended in the same volume of pure water. All the measurements were repeated for three times.

This delayed inactivation is also called residual inactivation effect, which is ascribed to hydrogen peroxide generated from the DBD treatment. To verify this, we measured the change of hydrogen peroxide vs. discharge time, as shown in Fig. 4. It shows that H_2_O_2_ concentration can be raised up to 8 mM in the DBD treated sample’s supernatant solution for discharge time at 8 min. With H_2_O_2_ produced in solution, the living cells can be continuously inactivated. Figure 5 shows the quantitative analysis of residual inactivation based on the flow cytometry assessment method (Supplementary Fig. S5 and Fig. S6). Figure 5(A) shows that the inactivation rate increases with the prolongation of delay time, where the samples were harvested with different DBD treatment time and delay time. Besides, it shows that when discharge time is less than 2 min, the residual inactivation effect is not pronounced. In addition, we also took the DBD treated algal cells out of the DBD-treated solution, re-suspended them in pure water, and then measured the residual inactivation rate, with the result presented in Fig. 5(B). It shows no significant change of inactivation rate with delay time, further confirming that H_2_O_2_ plays the role in the residual inactivation effect.

## Discussion

Generally, DBD treatment can induce both inactivation and repair mechanisms in biological systems. For example, Hou *et al.* observed the first decrease and subsequent increase in survival rate for helium-DBD treatment of *K. pneumoniae*^31^. So it is reasonable to assume that the residual effect may also include both residual inactivation effect and residual repair effect. However, the above results in Fig. 5 indicate that in our cases the residual inactivation effect must be dominant, especially for the DBD treatment with discharge time >2 min, for which relatively high concentration of H_2_O_2_ is produced (ca. 2 mM for discharge time 2 min according to the result of Fig. 4). Actually, in the inactivation experiment, to render effective residual inactivation, we set the DBD treatment time to be long enough to produce sufficient H_2_O_2_ so that it can continuously inactivate the remaining alive algal cells.

On the other hand, Fig. 5(A) also shows that with DBD treatment time being 6 min or longer, the increment of inactivation rate is already considerably reduced. This is because when most of the cells have been killed by DBD, the DBD treatment in later stage becomes not so efficient as that in the beginning stage. So in order to achieve more efficient inactivation effect, it is necessary to take advantage of the residual effect smartly rather than the simply prolonging the discharge time. In other words, to make full use of the DBD processing, the initial complete inactivation of algal cells is not necessary, and it is better to make use of the DBD-generated H_2_O_2_ to inactivate the remaining algae.

**Figure 6:**
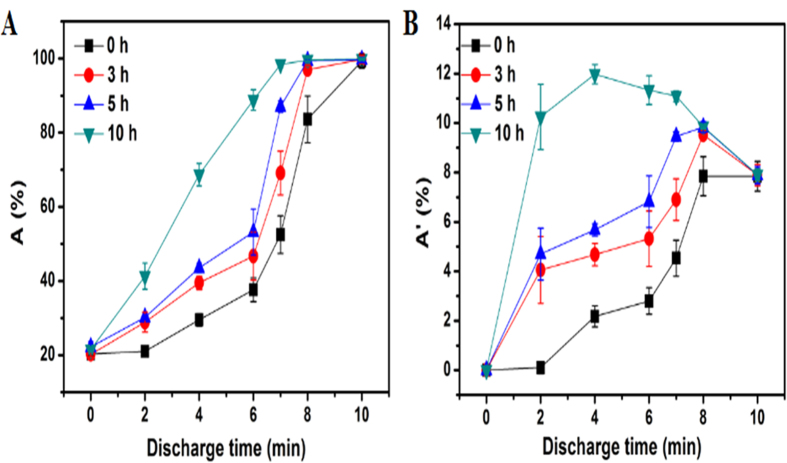
The inactivation rate (A) and unit time inactivation rate (A′) of *M. aeruginosa* for DBD treated different treatment and delayed times. The experiment was repeated for three times.

But, how to combine the virtues of DBD direct inactivation effect and its delayed inactivation effect, so that we can optimize the discharge conditions to achieve the economic and high efficiency DBD treatment? For this purpose, we tried to examine the residual effect more closely and determine the appropriate treatment time and delay time by defining the unit time inactivation rate, namely, A′ = A/T, where A is the total inactivation rate, T is the discharge time. Figure 6 shows the results of A and A′ which both change with the discharge time. However, different from A, A′ does not show a simple monotonic change with discharge time. Instead, it shows a maximum value which is also dependent on delay time. For example, for the 0 h delayed sample, the maximal A′ reaches at discharge time of 8 min; but for the 10 h delayed sample, 4 min of discharge time already ensures the maximal A′. So evaluation of the parameter A′ just suggests that it is not efficient to elongate the discharge time to achieve optimal inactivation effect, rather, balancing both the discharge and delay times properly can facilitate the best processing efficiency in terms of consumption of energy and time.

**Figure 7:**
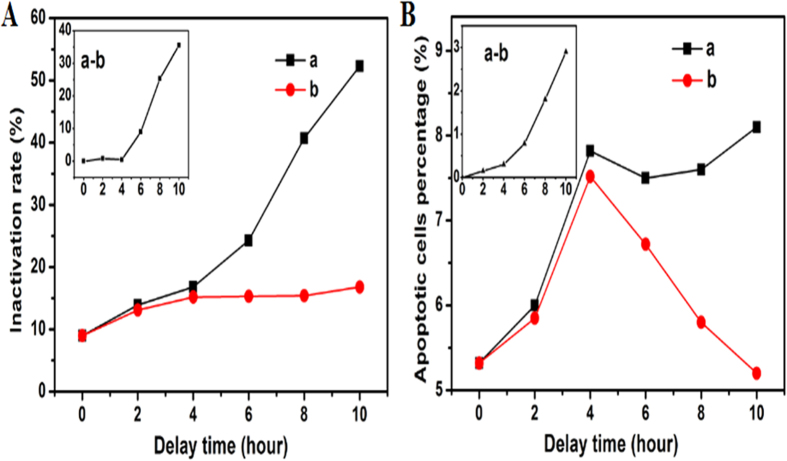
The variation of inactivation rate (A) and apoptotic cells (B) after DBD treated 3 minutes and delayed for different hours (a) the samples containing H_2_O_2_, (b) the samples discarding H_2_O_2_ and re-suspended in pure water.

Furthermore, we are also interested in the investigation of apoptosis of algal cell as observed with flow cytometry and confirmed by the apoptosis assays but essentially ignored in previous studies. The foregoing experiments prove that the H_2_O_2_ can cause the residual effect on *M. aeruginosa*, which includes both the direct inactivated dead cells and apoptotic cells. This result is consistent with the report by Ding^30^ that H_2_O_2_ can induce cell death and apoptosis. We also observed that the number of apoptotic cells also increased with delay time (Supplementary Fig. S7). This indicates that H_2_O_2_ plays a role in the apoptosis of *M. aeruginosa*. But it arouses the question whether all the apoptotic cells stem from extracellular H_2_O_2_ effect? To answer this question, we then carried out the following research, with the result as shown in Fig. 7. In the experiment, to exclude the H_2_O_2_ effect, we compared three kinds of samples: (a) the samples containing both algal cells and the DBD treated solution with the DBD produced H_2_O_2_; (b) the sample discarding the supernatant containing H_2_O_2_ and then re-suspended the algal cells with same volume pure water; and (c) the samples containing the untreated normal algal cells but suspended in the DBD treated sample’s supernatant. A schematic diagram for illustrating the preparation of these three kinds of samples is shown in Supplementary Figure S8. Then the three types of samples were examined for the residual inactivation effect, respectively. From the analysis of the samples (a, b and c), we can conclude that not only H_2_O_2_ contributes to the residual effect, but also the initial direct damage induced by DBD on the cells plays a considerable role. In the data analysis, the effect by sole H_2_O_2_ can be estimated by subtracting the inactivation efficiency of sample (b) from that of sample (a). Figure 7(A,B) illustrate the efficiencies of the direct inactivation and delayed inactivation vs. delay time, respectively. The insets in both Fig. 7(A,B) unambiguously demonstrate that more cells become dead and apoptotic without the presence of H_2_O_2_. This thus suggests that the initial even mild damage on the cells can also play an important role in the residual inactivation effect. Moreover, the comparison between the sample (a) and (c) reveals that the DBD treated sample is indeed more sensitive to H_2_O_2_ (Supplementary Fig. S9), which also verifies that the DBD-induced initial damage on the cells is partly responsible for the residual interaction effect.

One possible reason for the initial damage contribution is that additional reactive oxygen species (ROS) in the cells may cause the enhanced apoptosis/death of *M. aeruginosa*. Interestingly, Fig. 7(B) shows that the number of apoptotic cells first increased but then decreased in the residual effect measurement. The DBD-induced initial damage on the cells can give rise to accumulation of ROS inside the algal cells. To test our conjugation of the role of ROS in apoptosis, we also measured the ROS inside the cells and observed that the intracellular ROS increased and then decreased with delay time (Supplementary Fig. S10). Therefore, this similar trend of changes in ROS and number of apoptotic cell also suggests that the apoptosis is closely related to the ROS induced in the DBD-treated cells.

## Conclusion

**Figure 8:**
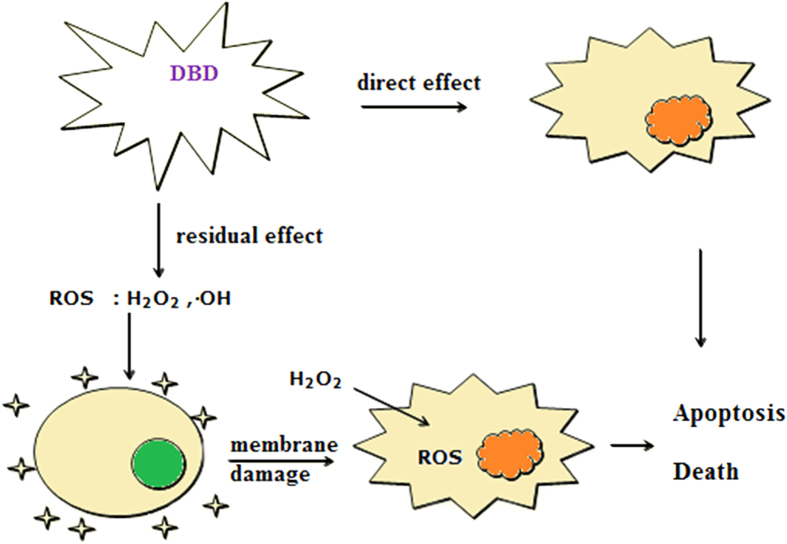
The schematic diagram for illustrating the inactivation effect and mechanism for the DBD treatment of algal cells.

In summary, we have scrutinized the DBD induced inactivation of *M. aeruginosa* by using the method of flow cytometry, which ensured sensitive and reliable assessment of algal status and population; with this we have distinguished and confirmed apoptotic cells caused by DBD plasma discharge, and unambiguously discriminated the residual effect apart from the direct inactivation effect. We have also confirmed that the residual effect is mainly due to the remaining H_2_O_2_ generated by DBD process, but the initial damage to the algal cells during the discharge also elicits intracellular ROS which can make a considerable contribution to the residual inactivation effect partly through the apoptosis mechanism. A schematic plot for explaining the inactivation effect by DBD treatment is illustrated in Fig. 8. Our focus on the residual inactivation effect in this work may therefore not only provide a better understanding of the DBD induced inactivation mechanism, but also facilitate us to optimize discharge conditions such as plasma treatment time and delay time, so as to further improve the DBD treatment efficiency and gain better control of algal blooms.

## Materials and Methods

### Experimental setup and apparatus

The schematic diagram of our DBD reaction apparatus is depicted as shown in Fig. 1. The main section used for treatment of *M. aeruginosa* consists of a reaction tank, which consists of a circular quartz plate cover with 1.5 mm thickness and 90 mm in diameter and a quartz container holding the *M. aeruginosa* solution. The reaction tank was put in the center of two stainless steel electrodes, with flowing gas filling the gap between the electrode and the surface of the solutions. For each experiment, 3 ml *M. aeruginosa* solution was added to the reaction tank. The distance between the upper electrode and the surface of the solution was 7 mm, and the depth of the cylindrical reaction tank was 8 mm, so the volume of the cylinder was about 22.6 ml.

The electrodes were connected to AC power supply. In this work, argon gas was introduced into the reactor before and during the discharge, and the flow rate was 0.5 L/minutes.

### Materials

Cyanobacterial species of *M. aeruginosa* (FACHB7806) were obtained from the Fresh water Algae Culture Collection of the Institute of Hydrobiology (FACHB), Wuhan, China. As dominant algae in eutrophicated water, *M. aeruginosa* was selected in this research work and cultivated in BG11 media. A series of 250 ml Erlenmeyer flasks containing 100 ml of the sterilized growth media were housed in an incubator at 25 °C under illumination on a 16-h light/8-h dark cycle after inoculating.

## Methods

### The measurement of inactivation rate of the *M. aeruginosa*

The inactivation rate of *M. aeruginosa* cells was investigated by the flow cytometric analysis. A FACSCalibur flow cytometer (Becton Dickinson, USA) equipped with an argon laser emitting at 488 nm was adopted for all fluorescent measurements. Non-algal particles were excluded from the analysis by gating on FSC/SSC/FL1/FL3. Data were collected and analyzed using CellQuest software (Becton Dickinson, USA). SYBR green I (Sigma) and propidium iodide (PI; Sigma) were used to determine the cells with intact and damaged membrane^32^. The green fluorophore of SYBR green I can permeate in live cell membrane and bind with DNA to make the cells give out green fluorescence, while the red fluorophore of PI can only permeate dead cell membrane and bind with DNA to make the dead cells give out red fluorescence. The working solution of the dyes were prepared according to Tao^33^: SYBR stock solution was diluted for 100 times by adding Milli-Q water to the stock solution of 1:10000 (v/v) and kept at −20 °C. PI stock solution was prepared by dissolving the solid powder in Milli-Q water as the final concentration was 1 mM and kept at 4 °C. 10 μL of both SYBR and PI stock solutions were added into the *M. aeruginosa* solution per milliliter at the same time, and then incubated for 15 minutes at room temperature in dark. After staining, we acquired 20000 cells for analysis. The green fluorescence of SYBR green I was determined by FL1, and the red fluorescence of PI was determined by FL3. All the flow cytometry data were exported by FlowJo software. For data analysis and drawing the academic figures we used Origin 8.0.

### ROS detection

The fluorescence probe 2′,7′—dichlorodihydrofluorescein diacetate (H_2_DCFDA) was used to detect the cellular production of reactive oxygen species (ROS)^34^ after DBD treatment. The final treatment concentration of H_2_DCFDA was 10 mM, and then incubated for 1 hour in dark at 37 °C. This method relies on the cellular esterase which can transform H_2_DCFDA into H_2_DCF, and then H_2_DCF can react with ROS to form the green fluorescence compound DCF which is thus detected by flow cytometer (Becton Dickinson, USA) through FL1.

### Determination of the Hydrogen Peroxide concentration

The concentration of the hydrogen peroxide after the DBD plasma treatment was measured spectrophotometrically at 410 nm after mixing with titanium sulfate in acidic condition^35^.

### TUNEL assay

One Step TUNEL Apoptosis Assay Kit (Beyotime, China) was used to detect the apoptotic cells in the *M. aeruginosa* solution after DBD treatment or after several hours of exposure to H_2_O_2_. Cells analyzed with TUNEL kit were fixed for 2 h with 2% paraformaldehyde in PBS at room temperature and then washed with PBS. Cells were permeabilised for 1.5 h in PBS containing 0.1% Triton X-100 and 0.1% sodium citrate at 4 °C^30^. The labeling and signal conversions were carried out according to the manufacture’s protocol. Then the samples were analyzed by the fluorescence microscope (Olympus, Japan).

### Caspase-3 activity detection

CaspGLOW Fluorescein Active Caspase-3 Staining Kit (Bio Vision, USA) was used to verify whether the intermediate state cells were apoptotic cells^30^. The kit was used according to the manufacturer’s instructions. After labeling, cells were washed twice in buffer, and then the samples were analyzed under a fluorescence microscope.

## Additional Information

**How to cite this article**: Li, L. *et al.* New insight into the residual inactivation of *Microcystis aeruginosa* by dielectric barrier discharge. *Sci. Rep.* **5**, 13683; doi: 10.1038/srep13683 (2015).

## Supplementary Material

Supplementary Information
