# Supplementary material for: New insight into the residual inactivation of Microcystis aeruginosa by dielectric barrier discharge
Source: Sci Rep. 2015 Sep 8;5:13683. doi: 10.1038/srep13683 (PMC4561898; doi:10.1038/srep13683)
Supplement: Supplementary Information [file srep13683-s1.doc]

**Supplementary information**

**New insight into the residual inactivation of *Microcystis aeruginosa* by dielectric barrier discharge**

Lamei Li^1^, Hong Zhang^1^, Qing Huang^1, 2*^

^1^ Key Laboratory of Ion Beam Bio-engineering, Institute of Biotechnology and Agriculture Engineering, Hefei Institutes of Physical Science, Chinese Academy of Sciences, 230031;

^2^ The University of Science and Technology, Hefei, 230026

* Corresponding author: Prof. Dr. Qing Huang (huangq@ipp.ac.cn).

1. **Effect of applied voltage, discharge time, delay time and working gas on the inactivation of *M. aeruginosa***
2. Effect of the applied voltage, discharge time and delay time

The inactivation rate is increased with applied voltage, as shown in Fig. S1.


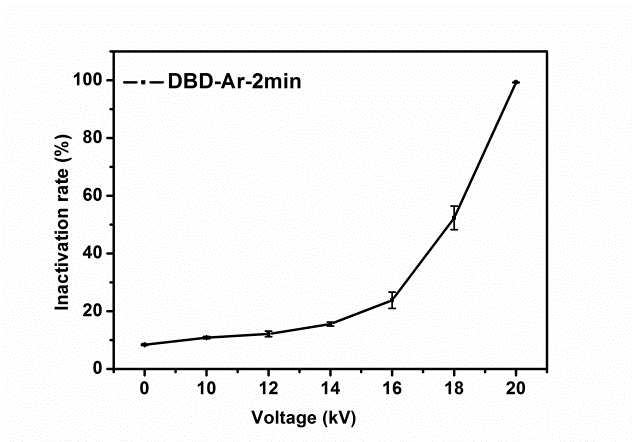


**Figure S1.** The inactivation rate of *M. aeruginosa* increases with applied voltage. The data shown in this plot were obtained with the argon-DBD treatment of 2 min.

1. Effect of working gas

Air-DBD is more efficient in inactivation of *M. aeruginosa* than argon-DBD, as shown in Fig. S3. However, the mechanism of inactivation of air-DBD is more complicated, and for the purpose of this study, we only focused on the inactivation effect induced by argon-DBD.


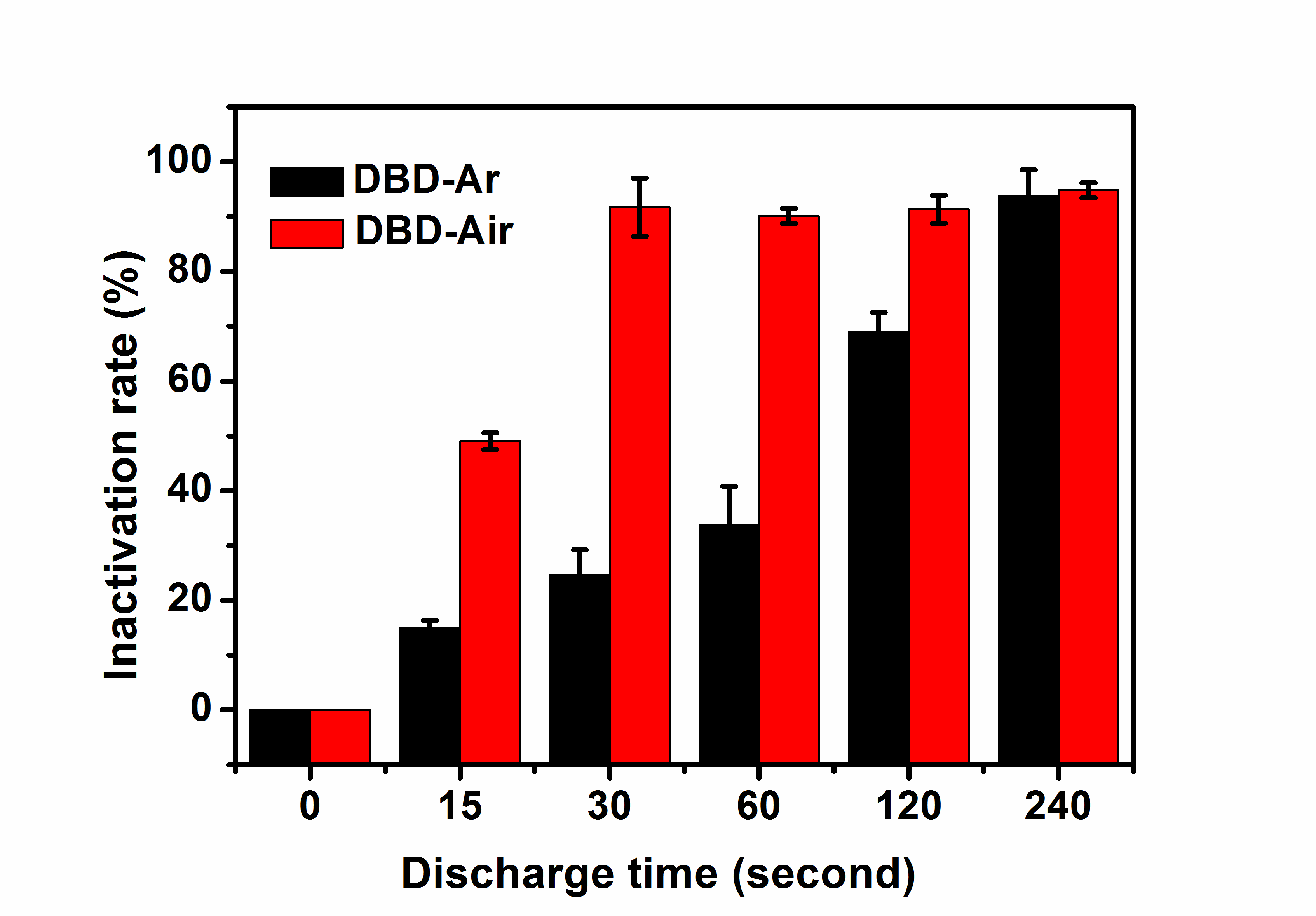


**Figure S2**. Comparison of the inactivation efficiency between argon-DBD and air-DBD.

**2. Identification of apoptotic cells induced by external H_2_O_2_ solely**

In our study, we utilized two apoptosis assay kits to verify the apoptotic cells, i.e., One Step TUNEL Apoptosis Assay Kit (Beyotime, China) for terminal deoxynucleotidyl transferase labeling (TUNEL) assay, and the caspase-3 activity assay kit. It was reported that 325 μM H_2_O_2_ could induce apoptotic-like cells after incubating 24 hours.[^5^](#_ENREF_5) So we incubated *M. aeruginosa* cells with 330 μM H_2_O_2_ for different hours, and the samples were harvested after 12 h, 24 h, 48 h exposure to H_2_O_2_. Then the samples were respectively examined by TUNEL assay and flow cytometry.


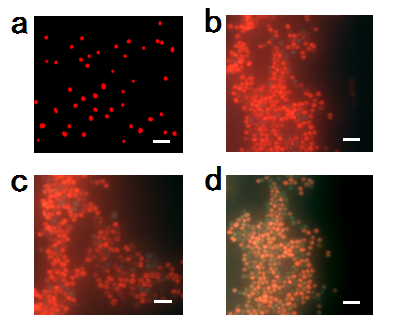

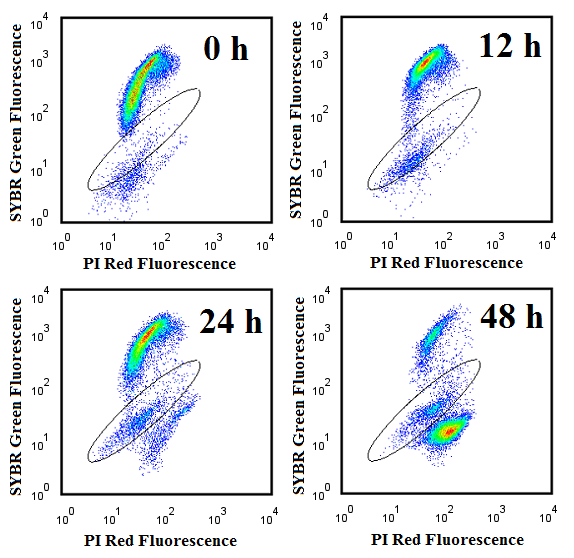


**Figure S3.** Detection of apoptotic cells by TUNEL assay using flow cytometry (a-d, cells exposure to 330 μM H_2_O_2_ for 0 h, 12 h, 24 h and 48 h, respectively. TUNEL-positive cells = green; others = red. Scale bar=10 μm).


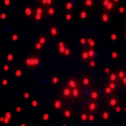
 A
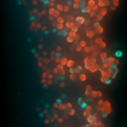
 B

**Figure S4.** Fluorescence microscopic images for identification of apoptotic cells using Caspase-3 activity kit (A: Control, B: cells exposure to 330 μM H_2_O_2_ for 48 h).

**3. The method of evaluation of inactivation effect through flow cytometry**

Formerly, the optical density at 680nm (OD_680_), an indirect index for cell viability, was measured to determine the inactivation efficiency.[^1-4^](#_ENREF_1) In this study, we employed the method through flow cytometry to determine the inactivation effect. In the flow cytometry experiment, the living/dead cells can be distinguished though two fluorescence dyes, namely, SYBR green I and PI. SYBR green I can permeate into living cell membrane and give out green fluorescence which can be detected by FL1 channel. PI can permeate into dead cell membrane and give out red fluorescence which can detected by FL3 channel.


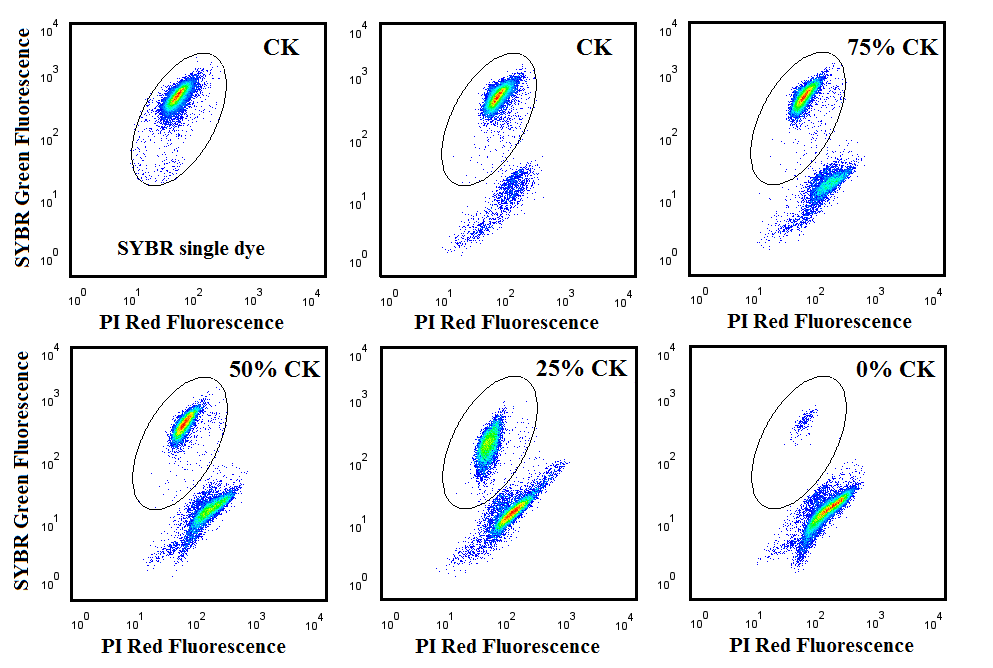


**Figure S5.** The flow cytometry results for mixture living cells and dead cells with different proportions. “CK” stands for “control check” which refers to the samples containing the nearly 100% of living cells.


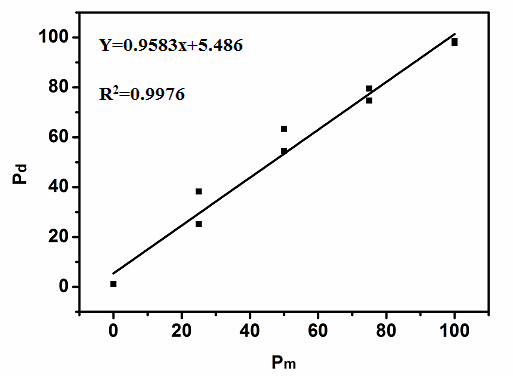


**Figure S6.** The fitting line of estimated value (P_d_) and proportion value(P_m_) based on the flow cytometry measurements shown in Figure S4. R^2^ value is 0.9776 for the fitting.

**4. Inactivation of *M. aeruginosa* depending on delay time**

The remaining H2O2 can continue to inactivate the algal cells, leading to increased numbers of both dead and apoptotic cells with delay time.


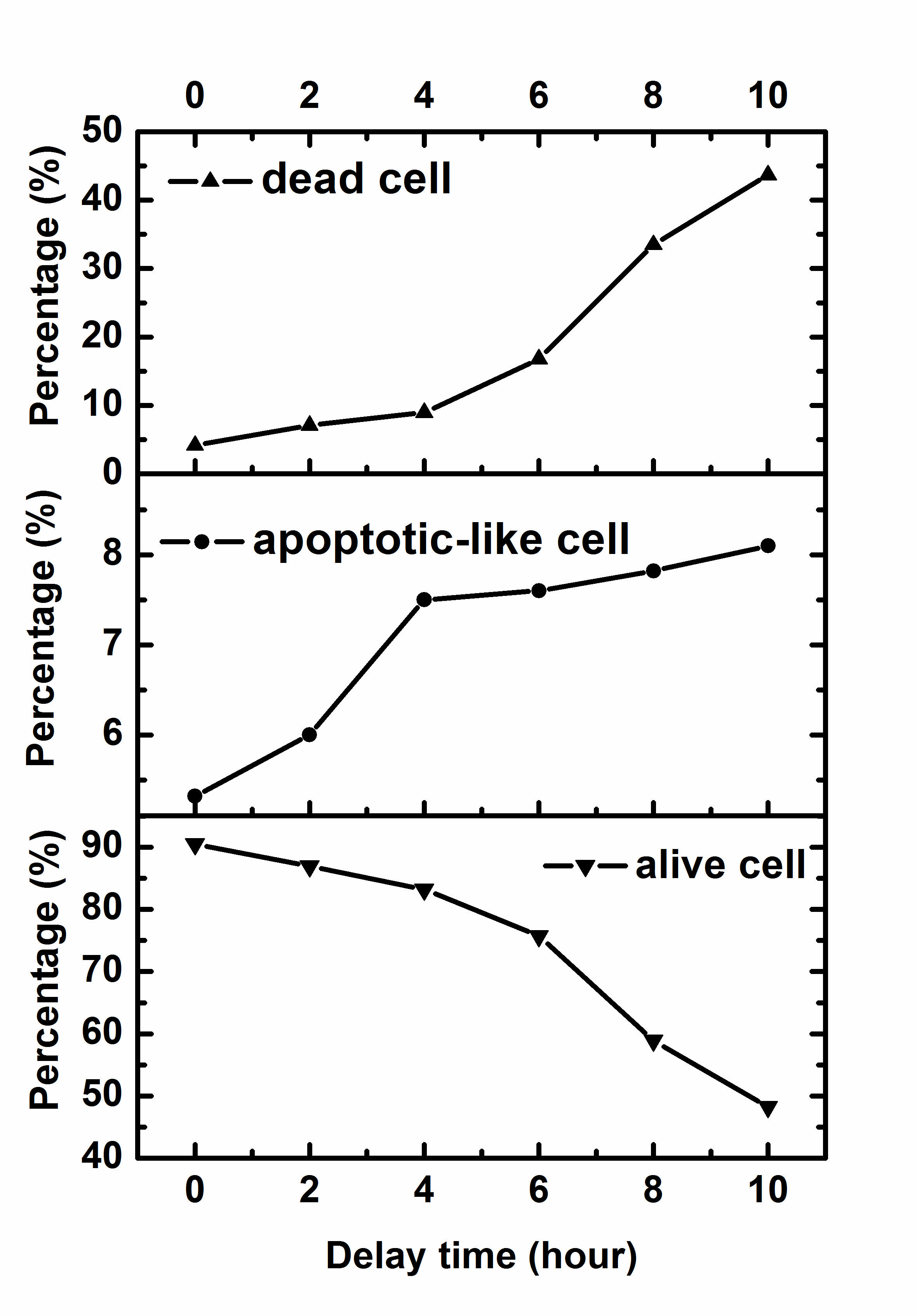


**Figure S7.** The populations of three states of the DBD treated algal cells (3 min of discharge time), which changes with the delay time after the DBD treatment.

**5. The three kinds of samples used for the evaluation of residual inactivation effect.**


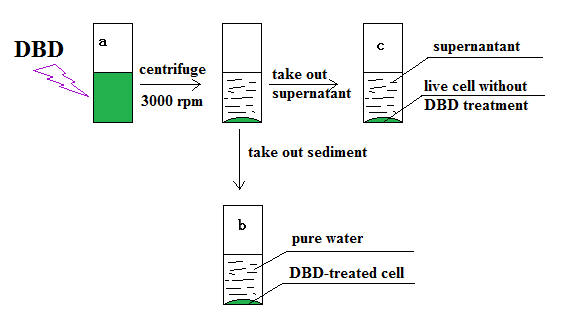


**Figure S8.** The schematic plot for illustrating the preparation of three different samples in the study.

**6. The effect of the DBD sample supernatant on the normal living cells.**


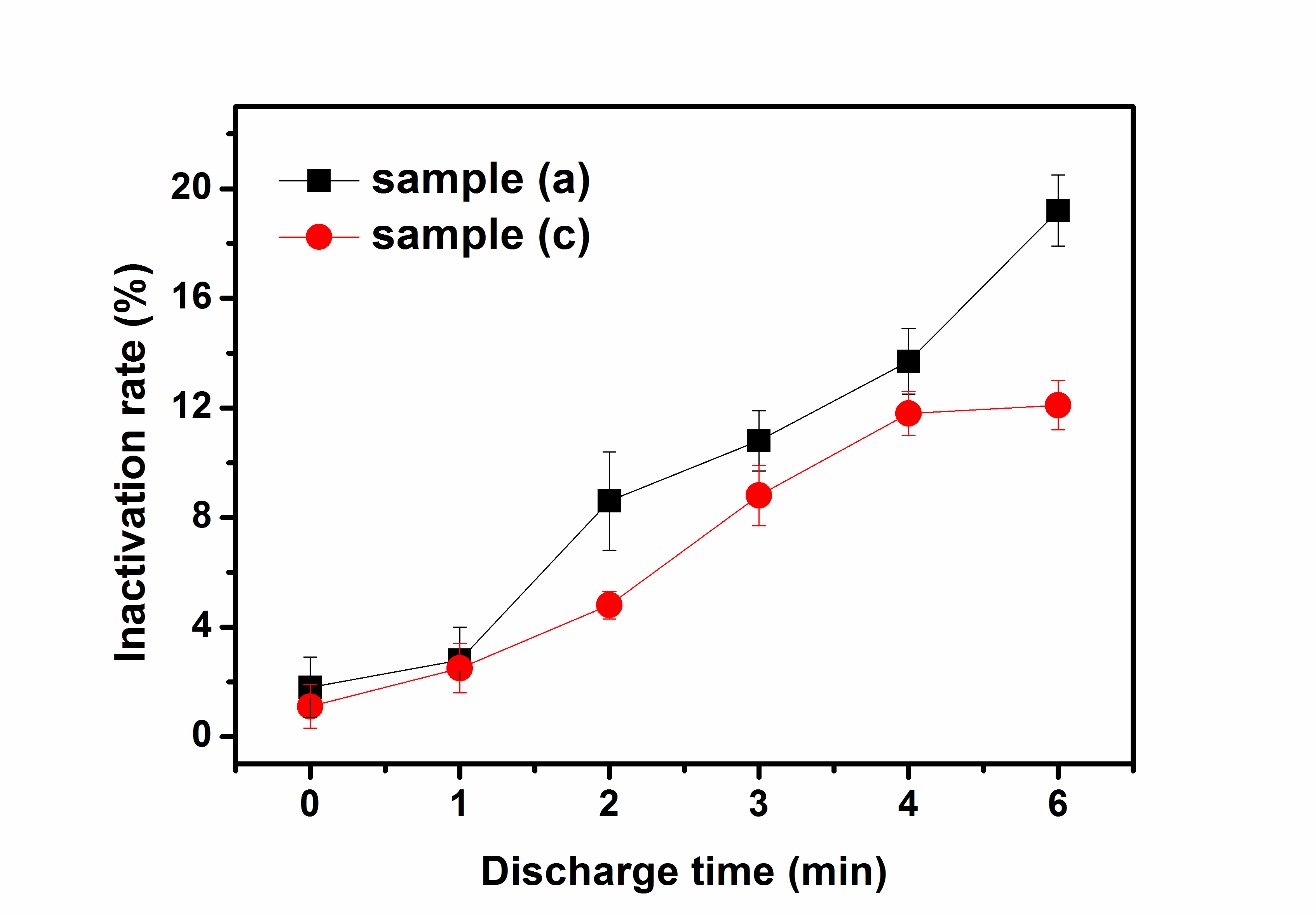


**Figure S9**. The comparison of samples (a) and (b) defined above as shown in Fig. S9. The inactivation rates were measured with delay time of 4 hours. It shows that the DBD-treated cells were more sensitive to H_2_O_2_ in the supernatant. This experiment suggests that the initial damage caused by DBD on the algae cells may also plays a role in the inactivation of the algal cells.

**6. Measurement of intracellular ROS level**


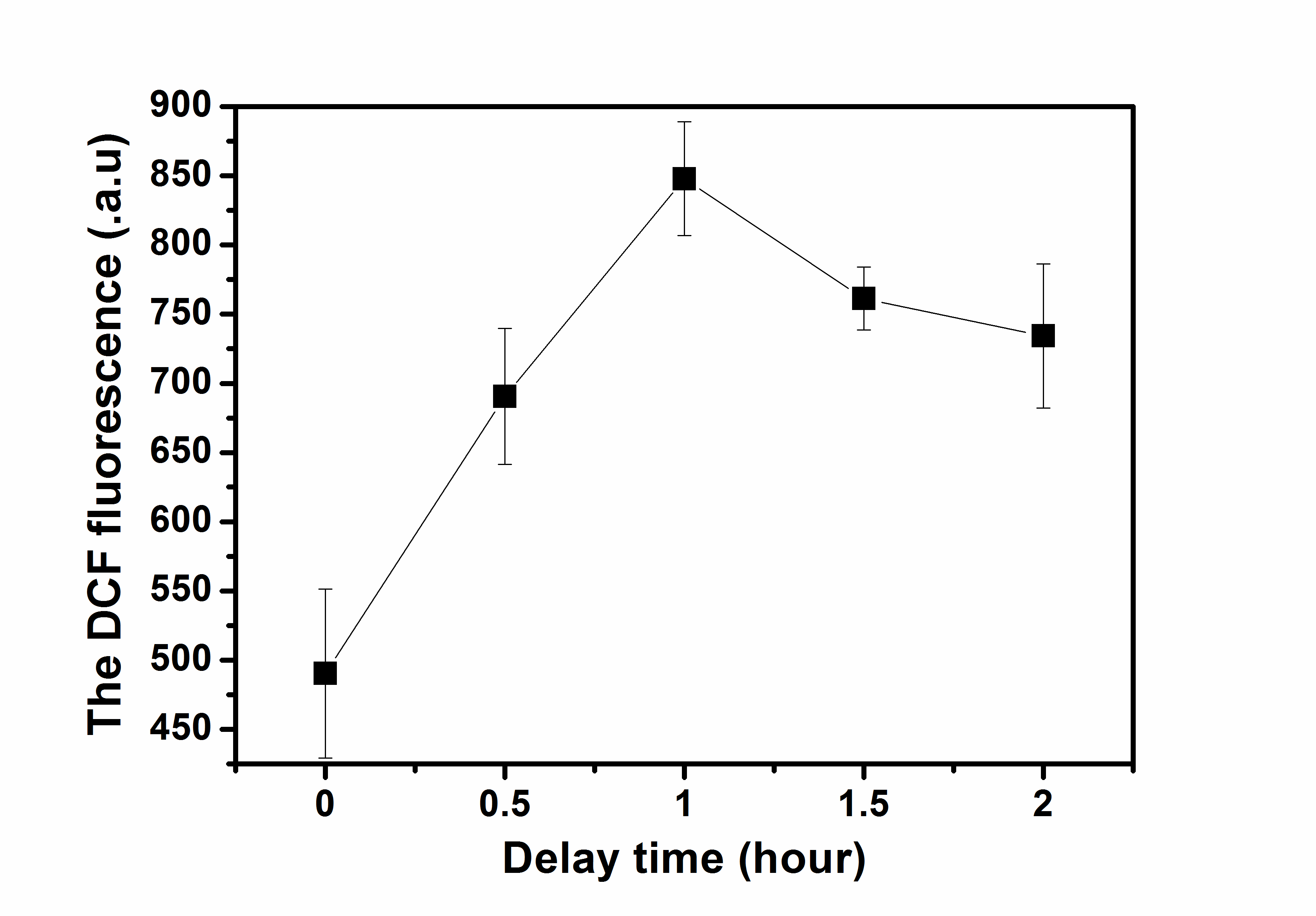


**Figure S10.** The intracellular ROS level assessed by DCF-fluorescence changes with the delay time (the measurements were repeated three times.)

1 Wang, C.-h., Wu, Y., Li, G.-f. & Shen, X.-q. Enhanced physical and chemical processes by solid packing in the plasma reactor for the inactivation ofMicrocystis aeruginosa. *Desalination and Water Treatment* **22**, 156-160, (2012).

2 Pu, S., Chen, J., Wang, G., Li, X. & Ma, Y. Inactivation of Microcystis aeruginosa using dielectric barrier discharge low-temperature plasma. *Applied Physics Letters* **102**, 194105, (2013).

3 Ma, B. *et al.* Influence of ultrasonic field on microcystins produced by bloom-forming algae. *Colloids Surf B Biointerfaces* **41**, 197-201, (2005).

4 Wang, C.-h., Wu, Y. & Shen, X.-q. A multi-wire-to-cylindrical type packed-bed plasma reactor for the inactivation of M. aeruginosa. *Journal of Electrostatics* **68**, 31-35, (2010).

5 Ding, Y., Gan, N., Li, J., Sedmak, B. & Song, L. Hydrogen peroxide induces apoptotic-like cell death in Microcystis aeruginosa (Chroococcales, Cyanobacteria) in a dose-dependent manner. *Phycologia* **51**, 567-575, (2012).
